# Polymer-Based Scaffold Strategies for Spinal Cord Repair and Regeneration

**DOI:** 10.3389/fbioe.2020.590549

**Published:** 2020-10-07

**Authors:** Wenrui Qu, Bingpeng Chen, Wentao Shu, Heng Tian, Xiaolan Ou, Xi Zhang, Yinan Wang, Minfei Wu

**Affiliations:** ^1^Department of Hand Surgery, The Second Hospital of Jilin University, Changchun, China; ^2^The Orthopaedic Medical Center, The Second Hospital of Jilin University, Changchun, China; ^3^Department of Biobank, Division of Clinical Research, The First Hospital of Jilin University, Changchun, China; ^4^Key Laboratory of Organ Regeneration and Transplantation of Ministry of Education, The First Hospital of Jilin University, Changchun, China; ^5^Department of Burn Surgery, The First Hospital of Jilin University, Changchun, China

**Keywords:** spinal cord injury, polymer-based scaffold strategy, neural protection, neural regeneration, function recovery

## Abstract

The injury to the spinal cord is among the most complex fields of medical development. Spinal cord injury (SCI) leads to acute loss of motor and sensory function beneath the injury level and is linked to a dismal prognosis. Currently, while a strategy that could heal the injured spinal cord remains unforeseen, the latest advancements in polymer-mediated approaches demonstrate promising treatment forms to remyelinate or regenerate the axons and to integrate new neural cells in the SCI. Moreover, they possess the capacity to locally deliver synergistic cells, growth factors (GFs) therapies and bioactive substances, which play a critical role in neuroprotection and neuroregeneration. Here, we provide an extensive overview of the SCI characteristics, the pathophysiology of SCI, and strategies and challenges for the treatment of SCI in a review. This review highlights the recent encouraging applications of polymer-based scaffolds in developing the novel SCI therapy.

## Introduction

Spinal cord injury (SCI) constitutes a considerable portion of the global injury burden is mainly caused by falls and road accidents (Wagner et al., 2018; David et al., 2019; Hutson and Di Giovanni, 2019). In 2016, the global number of prevalent cases of SCI was 27.04 million. SCI is a debilitating and irreversible injury leading to the complete or incomplete loss of sensory and motor function beneath the injury area, depending on the extent of the injury (Ordikhani et al., 2017). Such injury causes not only disabilities for individuals and their families, but also burden health systems and economies with loss of productivity and high health care costs. The lifetime cost of SCI approximately ranges between US$ 1 and 5 million per individual, depending on the level and severity of the SCI (Courtine and Sofroniew, 2019).

The subsidiary cascades remarkably enlarge the initial injury into neighboring tissues, as well as the spinal cord portions, followed by neuronal and glial wasting, scar and cavity formation, proliferation and activation of microglia and astrocytes, decreasing the extension of the subsidiary injury but also inhibit axon rejuvenation by developing chemical, as well as physical barriers to axon regrowth (Hutson and Di Giovanni, 2019). During the chronic phase, SCI patients usually have a neurological disability, mainly due to the disruption of spinal connectivity and failure of axon tracts about the lesion (Ahuja et al., 2016).

In the last decades, significant research advances have been made in increased comprehension of the SCI pathophysiology. The vital therapies for acute SCI aimed at preventing ongoing direct SCI and neuroprotection to reduce the secondary damage. At the same time, the treatments for chronic SCI center on neural rejuvenation, plasticity, and rehabilitation treatment while there were no effective conventional therapeutic attempts to restore mobility and sensation following SCI (Ahuja and Fehlings, 2016).

Even though significant advances have been made in the pathophysiology of SCI provides, many great promises have been made in improving therapeutic strategies for restoring function in animal models. The few approaches that have advanced to clinical trials have demonstrated insignificant or no efficacy in facilitating functional recovery (Hutson and Di Giovanni, 2019). Present therapeutic approaches for SCI are limited to, and pharmacotherapy, inadequacy, and complications of these therapies reveal that novel treatment approaches are needed (Hu et al., 2018).

**SCHEME 1:**
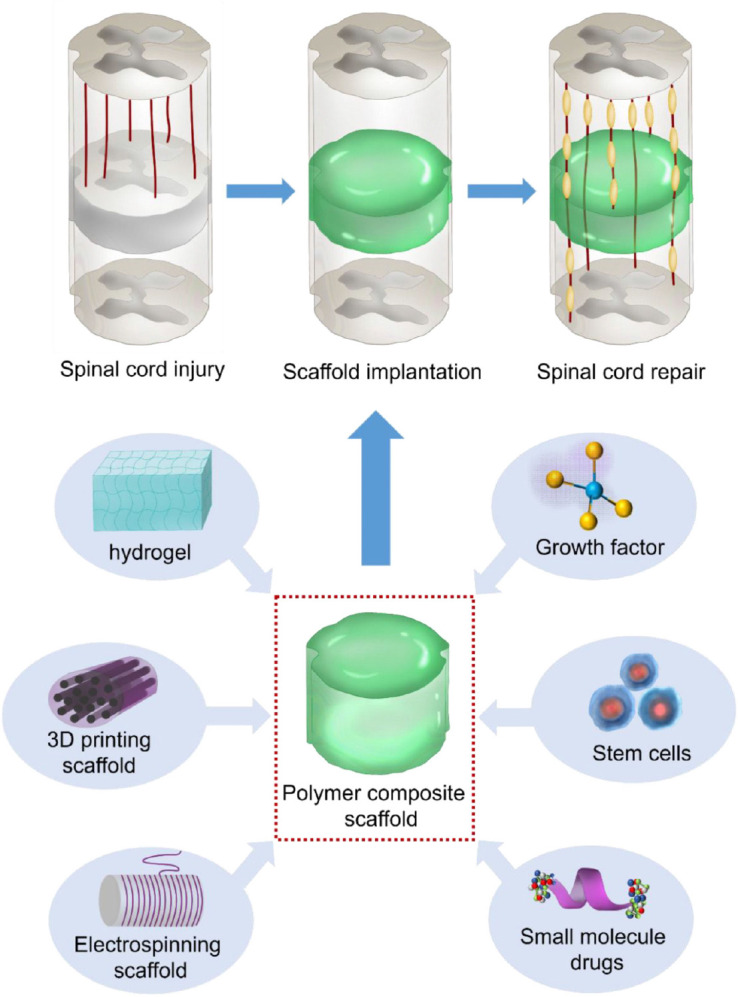
Schematic diagram of polymer composite scaffold repair for spinal cord injury.

The currents advancements in material science have resulted in the use of novel biomaterials to promote effective tissue regeneration following SCI. Biomaterial therapies are intended to regenerate the anatomic structure, as well as the function in combination with cells and/or active biomolecules (Chen et al., 2018; Qiao et al., 2019; Sun et al., 2019a; Yang et al., 2020). Recently, polymer-based materials have become a newly emerging strategy in SCI. The use of implantable polymer to enhance spinal cord rejuvenation intends to fill the created gap in the injured site and additionally modify the injured area toward a pro-restorative environment that provides physical cues for the rejuvenation of the axons (Ahuja and Fehlings, 2016). Based on these criteria, the polymer material must have applicable chemical, mechanical, as well as physical features for cell viability and tissue development. Because of the importance of this research topic, although there have been many similar review articles on the use of biomaterials for SCI (Liu et al., 2019; Zhang et al., 2019), there has not been a systematic summary and detailed classification of the materials and applications of polymers. Herein, we review the novelty of polymer therapy and in integrated treatments providing insightful possibilities for future study and prospective for safe clinical application (Scheme 1).

## Pathophysiology of SCI

### SCI-Induced Primary Injury

The phages of SCI pathophysiology include immediate, acute 60 (seconds to minutes), sub-acute (minutes to weeks), and chronic (months to years; Macaya and Spector, 2012). Physical strain to the spinal cord causes the vascular disorder, which leads to blood-spinal cord barrier rupture resulting in hemorrhage, edema, and ischemia, and subsequent invasion with immune cells, including neutrophils, as well as macrophages (Tran et al., 2018). Axonal and cellular membrane damage, axonal deterioration, and inflammation are prevalent at the beginning of the post-injury stage. Subsequently, proliferation and initiation of microglia and astrocytes follow. These cells produce several factors contributing to the resolution of the acute reactions, healing, and finally developing into a cavity bound by glial/fibrotic scarring. Neurological dysfunction and disappointing recovery are caused by axonal and cellular damage to spinal connectivity (Tran et al., 2018).

### Inflammation-Induced Subsidiary Injury

The primary injury results directly to an elongated subsidiary, which typically extends above and below the site of the lesion and can last for days or weeks. This secondary injury is characterized by the growth of the lesion epicenter tissue damage, including adjacent cell inflammation-mediated death of neighboring cells and axotomy of neurons survived in the primary trauma (Fitch et al., 1999). The extent of inflammation in the subsidiary injury is associated with the level of danger-associated molecular patterns, including endogenous alarmins, chromatin-associated protein high mobility group box1 (HMGB1), IL-1, S100, and histones. In this environment, immune cells become commonly activated to enter the M1 phenotype, which is typified by Inducible nitric oxide synthase (iNOS), reactive oxygen species (ROS), and reactive nitrogen species. Understanding the axis of molecular occurrences that happen soon after the injury can aid in an attempt to save as much nervous tissue through improved neuroprotective strategies (Tran et al., 2018).

### Glia and Fibrotic Scar After SCI

With the augmentation of inflammatory cells, oligodendrocyte progenitor cells (OPCs) and astrocytes become activated and under the scar formation. Though the scarring development aids in fixing inflammation reaction continued low-grade inflammation caused by macrophages, as well as the subsequent gliosis, reshapes the extracellular matrix (ECM) by increasing laminin, fibronectin, and collagen in the epicenter (Fernández-Klett and Priller, 2014). The exact genesis and role of these specific cells (i.e., pericytes, meningeal cells, which were referred to as fibroblasts) typically associated with connective tissue. Schwann cells emanating from the marginal roots migrate into the core of the lesion and also secrete fibroblast biosignatures and are closely related to deposits of laminin, fibronectin, and collagen (Zhang et al., 2013). At this phase, invading fibroblasts might even create a dense fibrous capsule that functions as a tissue barrier and interacts with the growth repressor biomolecules to further suppress axonal rejuvenation (Ordikhani et al., 2017). Transforming growth factor-beta (TGF-β) enhances astrocyte stimulation and following glial scar boundaries, also increases microglia/macrophage and fibronectin and laminin deposition (Logan et al., 1994). The positive function of glial scar in SCI reactions illustrates the need for a glial bridge in neural rejuvenation (Bloom, 2014). The glial component consists of NG2^2+^ oligodendrocyte precursors, reactive astrocytes, and microglia in the penumbra. The lesion penumbra around the fibrotic epicenter is cauterized by responsive glial cells and amplitude of chondroitin sulfate proteoglycans (CSPGs). Because of the elevated CSPG content in the glial scar, it is not astonishing that axon rejuvenation and plasticity are impeded after SCI. A crucial role of CSPGs in the adult central nervous system (CNS) constitutes the formation of the perineuronal net (PNNs), which of their potential to restrict plasticity in the CNS. Additionally, ECM biomolecules may escalate the rigidity of the bioenvironment, produce a physical barrier, as well as offer non-specific topographical cues; all of these can influence cellular movement. Interestingly, removal of suppressive ECM constituents, e.g., CSPGs, improves neurite proliferation.

The degradation and atrophy of axons in combination with the glial scar and cyst development poses a physical impediment to axonal rejuvenation after SCI (Eckert and Martin, 2017). Finally, the glial scar stabilizes about the cyst in the chronic phase, additionally surrounding the deteriorated axons because of the subsidiary injury.

## Strategies and Challenges for the Treatment of SCI

Regeneration after SCI involves purposed axon proliferation and remyelination of long tracts. The loss of the structural framework of the cord, including scar formation, impedes targeted axonal re-proliferation, as well as cell migration (Ahuja and Fehlings, 2016). After the SCI, non-neural cells, including perivascular fibroblasts, meningeal fibroblasts and pericytes could form the different size of lesion than can influence axon growth or regrowth. Astrocyte scar restrict the inflammation spread between the viable neural tissue and the non-neural cells which limit the axon regeneration. CSPGs are proteoglycans consisting of a core protein and glycosaminoglycan (GAG) side chains, such as aggrecan, versican, neurocan, and brevican, which are provably inhibitory to axon regeneration both *in vitro* and *in vivo*. Oligodendrocytes contain many potent axon growth-inhibitory molecules, which are release following the SCI, participate in the barrier to axonal repair and growth. Because there are no currently approved treatments for restoring the mobility and sensation after SCI, the present therapy approaches for acute SCI are restricted to surgical decompression, and intravenous high-dose methylprednisolone (MP) whose clinical efficacy remains unclear (Ahuja et al., 2016). Anti-inflammatory or neuroprotective approaches fail on the progression of the disease and the clinical outcomes.

Current therapies for SCI may primarily be classified into neuroprotective and neuroregenerative therapies. Neuroprotective treatments aim to avoid or prevent further advancement of subsidiary damage, while neuroregenerative therapies focus on restoring disabled function by repairing the disrupted neural circuit of the spinal cord (Ni et al., 2015). Nonetheless, once the period for treatment intervention to intercept subsequent damage events has elapsed, the focus turns toward the rejuvenation of the damaged spinal cord. Additionally, the treatment approaches aimed at alleviating or altering the progression of glial and fibrotic scar formation improve injury outcomes.

### Neuroprotective Strategies and Challenges in SCI

Following SCI, other inflammation founts may trigger blood-borne molecular signals, including LPS or cytokines, or activate immune cells that extend inflammation in the SCI lesions and aggravate tissue injury. Early inflammation following traumatic damage is advantageous and should not be prevented, but sustained inflammation has neurodegenerative potential. Neuroprotective treatments center on preventing or impeding the further advancement of the subsidiary injury through the alleviation of critical mechanisms, e.g., inflammation, apoptosis, or oxidative stress. Gene profiling shows up modulation and down-modulation of cell cycle-related gene expression linked to damage over the minutes, hours, and days following SCI, which might aid target future neuroprotective and restorative treatments (Di Giovanni et al., 2003). The timing of an anti-inflammatory or pro-inflammatory cell or an immune-mediated approach can, therefore, be crucial for neuroprotective strategies.

Methylprednisolone reduces the peroxidation of membrane lipids and post-inflammation, which consistently improve neurobehavioral outcomes. However, the use of MP is very controversial due to several side effects, such as gastrointestinal hemorrhage and wound infections. Other treatments include naloxone, tirilazad, and nimodipine showed efficacy in animal research, but clinical trials have failed to facilitate motor recovery. The glutamate receptor competitors or other kinds of repressions of the *N*-methyl-d-aspartate (NMDA) receptor were applied to protect neurons. Neurotrophins, including brain-derived neurotrophic factor (BDNF), facilitate functional recovery through regulating inflammatory cytokine levels (David et al., 2019). iNOS is a crucial pacifier of oxidative stress during neuroinflammation induced by SCI. Therefore, down-regulation iNOS could reduce nitric oxide-mediated cell death after SCI (Gao and Li, 2017; Maggio et al., 2017). Modulation of macrophage activity could regulate anti-inflammatory cytokine expression, restrict demyelination, and secondary damage from inflammatory reactions (David and Kroner, 2011; Shechter et al., 2013; Sun G. et al., 2019; Zhou et al., 2019, 2020).

BA-210 is a bacterial-derived toxin that impedes the Rho cascade of repressor proteins and subsequently enhances axonal proliferation. A phase trial was undertaken used the dura during surgery in 48 complete SCI patients (Fehlings et al., 2011). Rho is switched on by growth-repressor factors and modulates cascades that climax in the disintegration of the neuronal growth cone, injure of neural cells, and, eventually, the collapse of motor and functional restoration. Suppression of the switching of Rho is a potential therapy for injuries, including traumatic SCI. VX-210 represses Rho, was applied extramurally after decompression and stabilization surgery in a phase 1/2a study. The efficacy will be assessed in the future (Fehlings et al., 2018). Nogo-A is a protein to block axonal proliferation in the human CNS. Therefore, anti-Nogo is a monoclonal antibody engineered to target Nogo-A and facilitate neural rejuvenation. First-in-man intrathecal administration of neurite growth-enhancing anti-Nogo-A antibodies was administrated in acute SCI, showed functional recovery but needed further investigation (Kucher et al., 2018). Although potential, these drug agents have not yet to reveal efficacy in phase III trials.

### Neuroregenerative Strategies and Challenges in SCI

After SCI, the growth of the proximal axons encounters a hostile environment limiting spontaneous nerve rejuvenation (Hutson and Di Giovanni, 2019). This is supposed to be a result of the growth of suppressor factors that impede neuronal re-proliferation cascades and functional reconnections (Courtine and Sofroniew, 2019). Numerous factors need resolving to enhance axon rejuvenation in the SCI. In the complete SCI, there are two ways of reconnecting the links and functions beneath the injured area: bypassing the damaged region or reconstructing the functional tissue of the cysts and the scars. Neuronal survival, axonal proliferation, remyelination, and reconnection throughout the injured area are necessary for the repair of the spinal cord with the aid of linking grafts (Schwab, 2002). Tissue engineering provides the possibility of developing new therapeutic approaches for patients with polymer chemistry and cellular neurobiology (Chen et al., 2011).

The deterioration of neuronal tissue and the formation of a cystic cavity at the epicenter has made cell transplantation an exciting approach for SCI. Cell-based treatments, directly administered into the lesion region, are thought to facilitate tissue rebuilding and axonal rejuvenation by substituting injured or lost neural tissues. During the last decades, diverse lineages of cells have been applied, based on their unique functional capacities in the axon regeneration. The cell-based approach has become the essential aspects of translational medicine for SCI (Ni et al., 2015).

Schwann cells (SCs) supported axon regeneration in peripheral nervous systems, and they have particularly been reported to enhance axonal rejuvenation in the SCI (Fang et al., 2019; Ma and Liu, 2020; Marquardt et al., 2020). Olfactory ensheathing cells (OECs), Schwann cells, and marginal nerves can modify the microenvironment, which was favorable for neural regeneration. Stem cells, neural progenitor cells, induced pluripotent stem cells (iPSCs), embryonic stem cells (ESCs), mesenchymal stem cells (MSCs), etc. are all utilized for their pluripotent differentiation potential to substitute neuronal lineage cells, promote axonal rejuvenation and rebuild inter-neuron connections. Stem cells and neural progenitor cells are less tumorigenic relative to ESCs and were used safely to treat Parkinson’s disease, stroke, and cerebral palsy patients (Potter et al., 2008). iPSCs are anticipated to open enormous opportunities in the treatment of SCI, but it needs preclinical data to confirm the efficacy and safety of iPSCs (Okano et al., 2013; Lu et al., 2014; Strnadel et al., 2018). MSCs can be readily harvested from dental tissues, bone marrow, adipose, and blood. The effect of MSCs was more revealed in rodent studies when they were transplanted 1 week after damage, while no motor recovery at four months later (Syková et al., 2006). The crucial challenge is to obtain enough quantity of purified cells. Furthermore, SCI produces a hostile micro-environment that can impact on transplanted cell survival and integration. Nowadays, the optimal period for cell treatment is proposed as 7–10 days after injury. Direct transplantation of cells to the damaged site is often recommended, though this route could result in secondary injury. Clinically, a significant number of patients will undergo some natural neurologic recovery 4–6 months following the injury. Without controlled studies, it is unfeasible to distinguish whether improvements are linked to cellular transplantation or simply to the natural recovery ability of the patient. Generally, cellular transplantation is exclusively an investigational treatment.

### Strategies Targeting the Glial and Fibrotic Scar

The glial and fibrotic scars constitute a physical and chemical barrier to axon regrowth. Additionally, CSGPs and other repressor extracellular matrix biomolecules interact with receptors that inhibit axonal proliferation (Shen et al., 2009). Therefore, enzymatic digestion of CSPGs with chondroitinase ABC (ChABC) promotes axon rejuvenation, and functional restoration (Oudega et al., 2012). ChABC also facilitates neuroprotection by reducing pro-inflammatory CSPG stimuli (Bartus et al., 2014). Other therapeutic approaches, such as iron chelator, deferoxamine, and epothilone D, have feasible safety profiles in humans. Iron chelation could effectively reduce fibrotic ECM development. Epothilone D can have therapeutic effects due to microtubules’ axon stabilization (Sandner et al., 2018). Deferoxamine reduces total iron expression levels after SCI and impedes apoptosis and prevents glial scar formation (Chen et al., 2017).

A second treatment method aiming for the fibrotic and glial scars is the immature astrocytes into the damaged spinal cord. The transplantation of immature astrocytes into the lesion epicenter is another therapeutic method for the fibrotic and glial scar. Transplanted glial-restricted precursor cells, differentiated into oligodendrocytes, as well as astrocytes, alter the environment and reduce glial limitans development concurrent with supporting axon rejuvenation and sprouting (Hill et al., 2004).

Meanwhile, astrocyte progenitor transplantation promotes remarkable plasticity of rVRG-PhMN circuitry, reduction of macrophage response, and restoration of diaphragm function (Goulão et al., 2019).

Schwann cells are neuroprotective and promote axon regeneration and myelination, which were comprehensively studied as a strategy for SCI repair. Schwann cells improve motor recovery through attenuation of the activity for inflammasome complexes and associated inflammatory circuits (Mousavi et al., 2019).

Nevertheless, SCI remains to be a harmful condition with no cure. Cellular, rehabilitative, and molecular training and integrated therapies have shown potential results in animal models.

## Materials for Polymer Scaffolds

In recent years, a variety of materials, including natural and synthetic polymers, have been used in the preparation of scaffolds for SCI (Li et al., 2016).

### Natural Materials

Natural polymers mainly include hyaluronic acid (HA), collagen, chitosan, gelatin, silk protein, alginate, fibrin, laminin, fibronectin, agarose, cellulose, dextran, and compound. Here are some of the most commonly used natural polymers.

Hyaluronic acid is a long-chain polysaccharide and a crucial constituent of the extracellular matrix, HA, which can be enzymed into different molecular weights *in vivo* (Zarei-Kheirabadi et al., 2020). The physiological functions of high molecular weight HA and low molecular weight HA are completely different. High molecular weight HA can reduce the inflammatory response and fibrous scar formation after SCI through interaction with inflammatory cells and extracellular matrix proteins (Thompson et al., 2018). Low molecular weight HA (125–175 kDa) can promote angiogenesis. However, HA has poor cell adhesion and usually needs to be modified or compounded with other materials (Khaing et al., 2011).

Collagen is the main extracellular matrix component in various natural tissues, including central nervous tissue. Currently, more than 27 different subtypes have been found, of which type I collagen accounting for the largest proportion (Chen et al., 2017). Collagen has good histocompatibility, can promote cell adhesion and proliferation, support nerve adhesion, and growth. After SCI, collagen can be used for defect repair, which is beneficial to promote axonal regeneration. In addition, the gelation of collagen hydrogel *in situ* in the injured area makes it an excellent cell carrier and can maintain the transplanted cells to stay in the damaged part without being taken away by cerebrospinal fluid (Han et al., 2015).

Chitosan is a kind of positively charged biopolymer compound rarely seen in nature. It has abundant sources, low price, non-toxicity, non-irritation, non-antigenicity, heat-source reaction, non-hemolysis, non-mutagenicity, natural degradation, good histocompatibility, and controlled-release effects (Boido et al., 2019). In addition, during the treatment of SCI, chitosan has significant neuroprotective properties, maintains the integrity of the cell membrane, and can block the activity of lipid peroxidation (Elizalde-Peña et al., 2017).

Acellular matrix from the brain, spinal cord, and peripheral nerve tissue is also widely used to repair SCI (Tukmachev et al., 2016). After the nerve tissue is decellularized, extracellular matrix proteins are basically retained to provide proper nutrition for the restoration of the spinal cord, including important laminin and fibronectin, mucopolysaccharide, various collagen components, and GFs. In addition, it is in a sol state at low temperature and can be converted into a gel at body temperature, which is very convenient for injection use (Koèí et al., 2017).

### Synthetic Materials

In addition to natural materials, many synthetic polymers are also used for the preparation of SCI scaffolds, including polysialic acid (PSA), polylactic acid (PLA), polylactic-co-glycolic acid (PLGA), and polyethylene glycol (PEG) and so on, which can be individually designed according to needs to make its performance controllable (Nawrotek et al., 2017).

Polysialic acid is a class of linear, homogeneous α-2,8 unique carbohydrates linked to sialic acid (Guo et al., 2019). It is mainly attached to neural adhesion molecules in the vertebrate nervous system through typical *N*-linked glycosidic bonds. PSA regulates nerve cell development, nerve guidance, and synapse formation by changing the adhesion of nerve adhesion molecules in the nervous system, thereby playing a pivotal role in nerve development (Jung et al., 2017).

Polylactic acid is a novel kind of biodegradable material prepared by refining starch from renewable plant resources. PLA has excellent biocompatibility and has been extensively applied in biomedical engineering as bone nails, bone plates, and surgical sutures. The surgical line constituting PLA can be slowly hydrolyzed to lactic acid and metabolized by the body without removing the thread. The general decomposition period takes half a year to 1 years. In the use of SCI, macroporous scaffolds with directional channels are often prepared and implanted into adult rat SCI models.

Polylactic-co-glycolic acid is formed by randomly polymerizing PLA and polyglycolic acid (PGA). PLGA is a degradable functional polymer organic complex with improved compatibility, non-toxicity, good encapsulation, and film-forming properties and is extensively utilized in pharmaceutical, biomedical engineering biomaterials, and modern industrial fields (Ding and Zhu, 2018). In the United States, PLGA passed the Food and Drug Administration (FDA) certification and was officially included in the United States Pharmacopeia as a pharmaceutical excipient (Martins et al., 2018).

Polyethylene glycol is non-toxic, non-irritating, has excellent lubricity, moisture retention, dispersibility, water-solubility, and has better biocompatibility with many organic components (D’souza and Shegokar, 2016). In the treatment of SCI, PEG can inhibit the generation of free radicals, lipid peroxidation, and reverse the increase in cell membrane permeability (Liu et al., 2017). In addition, it can be used as a sealant to damage the axon membrane and has become a new method to repair the damaged nerve membrane.

## Forms of Polymer Scaffolds for Spinal Cord Injury

The research on SCI focuses on multiple aspects, including mechanism research, stem cell therapy, genes therapy, drug screening, and tissue engineering. Among them, tissue engineering has an irreplaceable advantage for the treatment of SCI (Vismara et al., 2017). Tissue engineering scaffolds can build nerve bridges between injury sites, which can provide a good microenvironment to guide axon contacts at both ends of the injury, and inhibit the formation of nerve scars to promote spinal cord repair (Li et al., 2016). In recent years, with the rapid development of materials science and preparation technology, different forms of scaffolds with different functions have been developed and used for spinal cord repair. Among them, hydrogels (Führmann et al., 2016), electrospun fibers (Schaub et al., 2016), and 3D printed stents (Koffler et al., 2019) are widely used. In addition, tissue engineering scaffolds can also be loaded with bioactive molecules and stem cells to further promote spinal cord repair (Oliveira et al., 2018).

### Hydrogels

Due to the high water content and variable mechanical properties similar to spinal cord tissue, high permeability, biocompatibility and degradability, hydrogels are focused on as the implants for SCI, which can meet the material exchange, metabolism and adhesion of nerve cells and be used as a carrier for various seed cells and bioactive molecules (Assunção-Silva et al., 2015; Boido et al., 2019).

### Materials for Hydrogel

Hydrogels have been widely used in regenerative medicine (Li et al., 2018; Qiu et al., 2020). In recent years, a variety of materials including natural and synthetic polymers have been used in the preparation of hydrogels for SCI (Li et al., 2016). Natural polymer mainly includes HA, collagen, chitosan, gelatin, silk protein, alginate, fibrin, laminin, fibronectin, agarose, cellulose, dextran, and their compound. Here are some of the most commonly used natural polymers.

Hyaluronic acid is a long-chain polysaccharide and an important component of extracellular matrix, HA, which can be enzymzed into different molecular weights *in vivo* (Zarei-Kheirabadi et al., 2020). The physiological functions of high molecular weight HA and low molecular weight HA are completely different. High molecular weight HA can reduce inflammatory response and fibrous scar formation after SCI through interaction with inflammatory cells and extracellular matrix proteins (Thompson et al., 2018). Low molecular weight HA (125,000–175,000) can promote angiogenesis. However, HA has poor cell adhesion and usually needs to be modified or compounded with other materials (Khaing et al., 2011).

Collagen is the main extracellular component in various natural tissues, including central nervous tissue, and more than 27 different subtypes have been found, with type I collagen accounting for the largest proportion. Collagen has good histocompatibility, which can promote cell adhesion proliferation and support nerve adhesion and growth. After SCI, collagen can be used for defect repair, which is beneficial to promote axonal regeneration. In addition, the gelation of collagen gel *in situ* in the injured area makes it an excellent cell carrier, which can maintain the transplanted cells to stay in the damaged part without being taken away by cerebrospinal fluid.

Chitosan is a kind of positively charged biopolymer compound rarely seen in nature. It has abundant sources, low price, non-toxicity, non-irritation, non-antigenicity, heat-source reaction, non-hemolysis, non-mutagenicity, natural degradation, good histocompatibility, and controlled-release effects.

Acellular matrix from brain, spinal cord and peripheral nerve tissue is also widely used to repair SCI. After the nerve tissue is decellularized, extracellular matrix proteins are basically retained to provide good nutrition for the repair of the spinal cord, including important laminin (LN) and fibronectin (FN), mucopolysaccharide (GAG), various collagen components and GFs. In addition, it is in a sol state at low temperature and can be converted into a gel at body temperature, which is very convenient for injection use.

Most of the synthetic polymer are biodegradable, including acrylic acid and its derivatives [such as polyacrylic acid, poly(*N*-isopropylacrylamide), polyacrylamide, etc.], PGA, PLA, polyphosphazene, and PEG and so on, which can be individually designed according to needs to make their performance controllable (Nawrotek et al., 2017).

### Crosslinking Mechanism of Hydrogel

**FIGURE 1:**
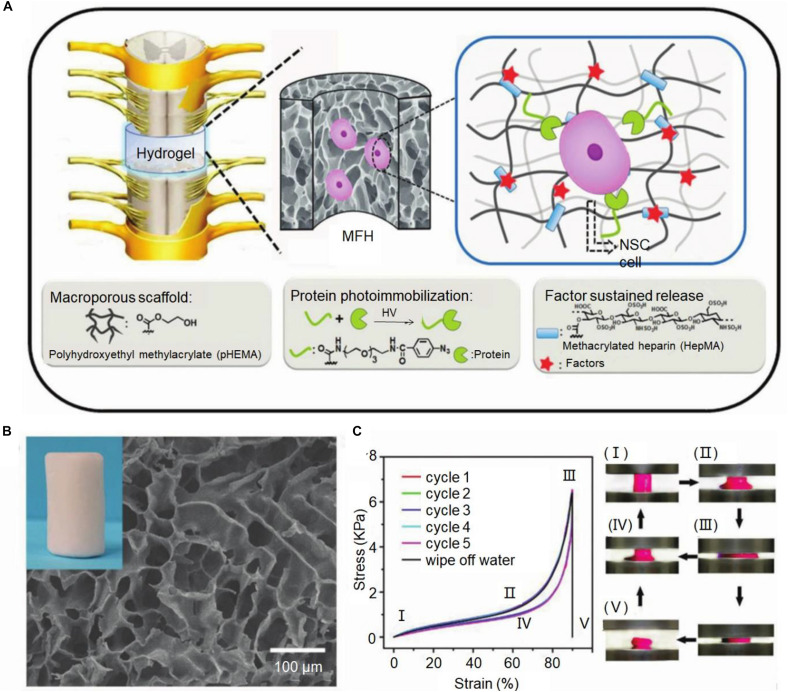
Macroporous functional hydrogel (MFH) scaffold for SCI. **(A)** Diagram of MFH scaffold implantation, protein photoimmobilization, and slow release of growth factors. **(B)** Scanning electron microscopy (SEM) image of MFH-2. **(C)** The cyclic compression stress-strain curves of this MFH scaffold. Reproduced with permission from Cai et al. (2019).

Hydrogel has a variety of classification method. According to different crosslinking methods, the hydrogel is usually divided into two broad categories: physical crosslinking hydrogels and chemical crosslinking hydrogels. Physical crosslinking hydrogels mainly by entanglement or non-covalent bond between the polymer chain, the hydrogen bond and the feeling of water interactions such as ion formation, such as physical junction between the polymer chain is not a permanent connection point, has the dynamic characteristic, but enough to maintain the integrity of the hydrogels in the water (Ma et al., 2016). Cai et al. (2019) used photochemical methods to prepare hydrogels that simulated the complex and dynamic properties of the extracellular matrix. The lyophilized hydrogel exhibited a sponge-like macroporous network structure interconnected with each other, and at the same time, the macroporous structure gave the hydrogel a wide range of flexibility and shape recovery performance and compression resistance (Figure 1; Cai et al., 2019). Chemical cross-linked hydrogels are formed by initiating copolymerization or polycondensation reactions using traditional synthetic methods or photopolymerization and radiation polymerization. The structure of chemical cross-linked hydrogels is relatively stable, with high strength, good reaction controllability, and easy operation. However, other substances are required to participate in the crosslinking process, accompanied by chemical reactions, which may affect the cell state.

### 3D-Printing Scaffolds

The complex innate structure of the spinal cord is considered to be another important reason for the difficulty in repairing after injury. In this case, mimicking the inherent microstructure of the spinal cord provides another direction for the treatment of SCI. Although biomedical scaffolds have been widely used for nerve repair, the lack of customized structures hinders their use *in vivo*. Current bioprinting functional organization methods lack proper biomanufacturing techniques to construct complex three-dimensional microstructures. Therefore, in the past decade, 3D printing technology has received extensive attention.

Generally, 3D printing refers to the geometry of a specific process of adding specific materials or curing to form a 3D object under computer control. 3D bioprinting refers to the use of 3D printing technology to combine cell GFs and biological materials to produce natural features that mimic biomedical ingredients. 3D printing technology can design the structure and performance of the stent, control the mechanical properties of the stent through the pore structure, and adjust the biological activity and degradation of the stent through the chemical composition. In addition, 3D bioprinting allows the customization of complex and complex structural carbon nanomaterials at the micron level, while advanced biomaterials (such as conductive polymers) are currently used as bio-inks in many printing systems.

### Technology of 3D Printing

Inkjet biological printing is an economic and effective technology adopts the control method of ink-jet printing technology can be well dispersed biological ink it allows and non-contact deposited at the same time the micron resolution cells in some direction in the technology is not high due to the biological ink viscosity, often leads to low mechanical properties of the structure. Inkjet printers contain thermosensitive and piezoelectric types. FDM technology uses filaments made from thermoplastic polymers to construct 3D structures. With the application of temperature, the filaments are heated in the nozzle to obtain a printable form (semi-liquid) and extruded onto the platform. Stereo lithography consists of ultraviolet lasers that focus on a hydrogel or resin that is photosensitized by the addition of a photoinitiator.

**FIGURE 2:**
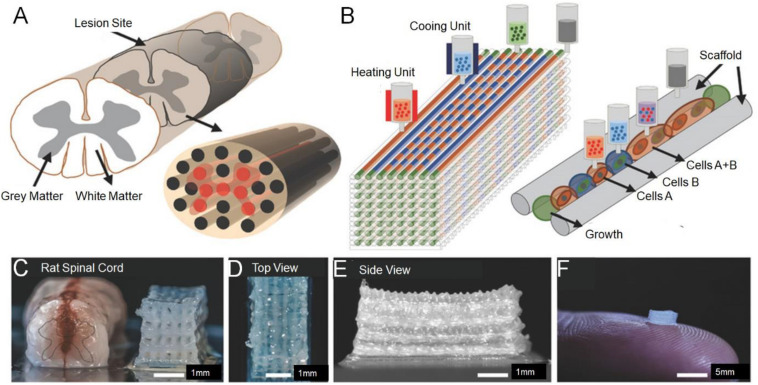
Experimental methods for 3D printing spinal cord scaffold. **(A)** Schematic of the anatomical structure of spinal cord in cross section showing gray matter, along with the white matter periphery, and the 3D-printed multichannel scaffold for modeling the spinal cord. **(B)** Schematic diagram of the layer-by-layer 3D printing process. **(C)** Comparative photograph of rat spinal cord cross-section and multi-channel 3D printing spinal cord scaffold. The number of channels could be adjusted according to the size of the required bracket. A top view **(D)** and the side view **(E)** of the scaffold. **(F)** A 2 mm × 2 mm × 5 mm sized scaffold on top of a finger showed the scale of the scaffold. Reproduced with permission from Joung et al. (2018).

Koffler et al. (2019) report the use of a method of continuous Projection printing method electronic Scale to create a complex central nervous system structure for application in spinal cord regeneration medicine. The 3D printing technology of multi-walled carbon nanotube (MWCNT)-hydrogel composite nerve scaffolds with porous structure, adjustable and well dispersed can easily produce complex 3D scaffolds with extremely complex microstructure and controllable porosity (Lee et al., 2018). In one study, a new 3D printing technology was used to produce a scaffold with a designed structure. Meanwhile, collagen and chitosan composite materials were used for compatibility and strength balance (Sun et al., 2019b). Joung et al. (2018) used 3D printing approach to precisely place iPSC derived spinal cord neuronal progenitor cells (NPCs) and OPCs in a biocompatible scaffold. The bio-printed spinal cord neuron progenitor cell (sNPC) differentiates, and axons extend in the micro-scale scaffold channels, as well as the bioactivity of the neuronal network were verified by physiological spontaneous calcium flux studies. The platform could be utilized to prepare new biomimetic hydrogel scaffolds, simulate complex central nervous system tissue structures *in vitro*, and design new clinical methods for the treatment of neurological diseases, including SCI (Figure 2; Joung et al., 2018).

### Electrospun Fibers

**FIGURE 3:**
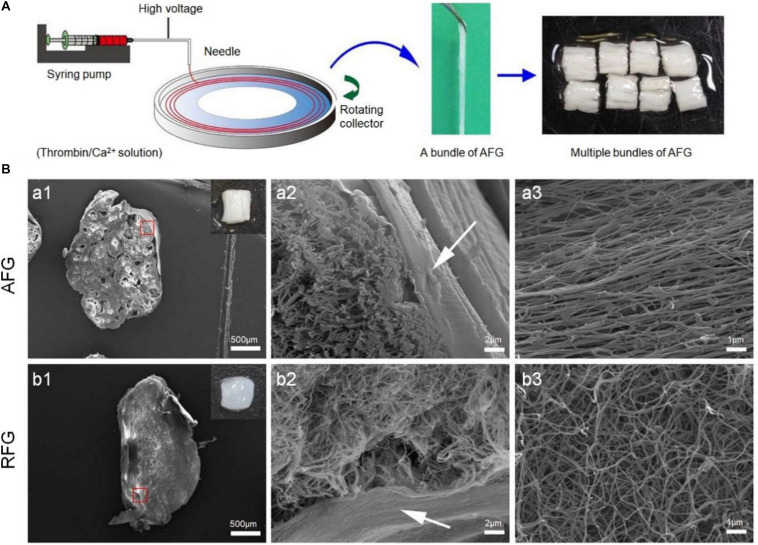
Aligned fibrin hydrogel (AFG) scaffold for rat T9 cerebrospinal cord injury. **(A)** AFG design procedures. **(B)** SEM images of the cross-sections of AFG and random fibrin hydrogel (RFG) supports and respectively show the layered oriented structure of AFG and the random fiber structure of RFG. (a1) and (b1) are low magnification SEM images of cross sections of AFG and RFG respectively, and the insert pictures are general photographs of the scaffolds respectively. (a2) and (b2) are high magnification SEM images of AFG and RFG scaffolds respectively, and the arrows are the alginate layers. (a3) and (b3) are SEM images of longitudinal sections of AFG and RFG scaffolds. Reproduced with permission from Yao et al. (2018).

The function of the axon is to transmit the action potential of the cell body to the synapse of the nervous system, and it is the main channel for nerve signal transmission. In the process of signal transmission in the spinal cord, a large number of axons participate together and become bundled, called nerve fibers. Axon regeneration is of great significance to the restoration of nerve function. However, after the SCI, the injury gap and glial scars made it difficult to find and reconnect the axons. Electrospinning technology has been widely used in the field of tissue repair because it can easily produce fibers with high surface area to volume ratio (Ding et al., 2019). Electrospinning is a process, which uses electric fields to generate fine fibers using synthetic materials or biomolecules (Ding et al., 2019; Feng et al., 2019; Wang C. et al., 2019). Electrospinning can produce oriented matrices and prepare ideal bridge prostheses, which can affect the axon orientation and growth of the array. In addition, by improving the electrospinning equipment and technology, a variety of fiber forms were stimulated, including twisted, core-shell, hollow, porous, and side-by-side multilayer surface structures. To simulate the natural environment of spinal cord tissue, Yao et al. (2018) prepared 3D layered fibrin hydrogels with directional structure and hardness using electrospinning and molecular self-assembly. The electrospun aligned fibrin hydrogel (AFG) fibers were stacked into 2 mm wide bundles and then cut into 4 mm long lengths to fit the SCI injury gap. It is worth noting that the gross image of the assembled AFG scaffold was like that of the spinal cord. The elasticity of AFG and random fibrin hydrogel (RFG) was identical to that of the ECM of nerve tissue, which was 1.57 ± 0.11 kPa and 0.36 ± 0.02 kPa, respectively (Figure 3; Yao et al., 2018).

## Application of Polymer Scaffolds for Spinal Cord Injury

Minimizing secondary damage as much as possible to avoid nerve cell apoptosis can promote the repair of the spinal cord to the greatest extent. Due to its biocompatibility, biodegradability, porosity and mechanical properties, the engineering three-dimensional scaffold can provide a good physical environment for nerve regeneration. However, scaffolds without secondary injury inhibitors, bioactive molecules or seed cells have limited therapeutic effects. In response to this problem, a variety of polymers, including polymer stem cell scaffolds and polymer drug scaffolds, have been developed on scaffold composite platforms to promote the regeneration of injured spinal cords from different angles.

### Polymer Scaffolds Stem Cells Composite Platform

Stem cells can not only replicate themselves, but also have the multi-directional differentiation potential of stem cells. The stem cells have the latent energy of multiple differentiation and play the role of replacing the degeneration necrotic cells; stem cells can secrete anti-inflammatory cytokines, which inhibit the inflammatory response of the lesion microenvironment; dry cells can produce many cytokines GFs and cell adhesion factors, which play a very important role in improving the microenvironment and promoting tissue regeneration. They can produce various tissue cells, especially specific nerve cells. Stem cells after transplantation can also secrete a variety of cytokines to nourish nerves, inhibit secondary injury, strengthen angiogenesis, and thus improve SCI. However, the survival rate of stem cell transplantation alone is low, and the repair effect on the spinal cord is limited. In recent years, the combination of engineering three-dimensional scaffolds and stem cells has become an effective management strategy for spinal cord repair. Stem cell-bearing biological scaffolds can be implanted into damaged sites to improve stem cell survival and differentiation. In particular, various types of stem cells are loaded on different biological scaffolds to promote the differentiation of stem cells into neurons, increase the release of GFs, improve axon regeneration, fill the cavity with axon myelin and reduce scar formation. At present, a variety of stem cells including neural stem cells (NSCs), bone marrow MSCs, bone marrow MSCs are widely used in the research of repair of SCI.

Neural stem cells are those that exist in the nervous system and have the potential of differentiation. In summary, NSCs have the following properties: (1) Self-sustaining. The characteristics of stem cells are maintained in daughter cells. (2) Self-renewal and proliferation. It can continuously divide. (3) Multiple differentiations. It can differentiate into neurons, astrocytes, and oligodendrocytes. (4) The ability to respond to diseases and injuries. After neural tissue injury, NSCs can generate neural cells through suitable activation, differentiation, and maturation to establish new neural networks. In the spinal cord, has been found that are arranged in the central tube of ependymal cells *in vitro* has the ability to differentiate into neurons and glial cells, they are defined as NSCs, however, compared with the NSCs in the brain, most of the ependymal cells differentiate into astrocytes, not observed neurogenesis after SCI. In addition, in response to injury, these generated becomes with reactive astrocytes, the final formation of glial scar.

Induced pluripotent stem cell-derived sNPCs and OPCs were precisely placed in a 3D-printed biocompatible scaffold. Bio-printed SNPC differentiated and extended axons in microscale scaffold channels, and the activity of these neural networks was confirmed by physiological spontaneous calcium flux studies (Joung et al., 2018). Is tested in this study, the bridge, including adult neural stem/progenitor cells contained in methyl acrylamide chitosan (MAC) hydrogel and chitosan conduit to protect the interferon and platelet sources of growth factor-aa (PDGF-aa) produced by restructuring and biotin labeled n end is fixed on the MAC chain mildew affinity is not functional, induced neuron system or less sudden cell line respectively (Li et al., 2016).

Bone marrow stromal sell (BMSC) can produce a variety of neurotrophic factors, such as nerve growth factor (NGF plays), vascular endothelial growth factor (VEGF) and so on, in the process of repair of SCI patients, the above factors can regulate the neurotransmitter generated, adjust the survival of neuron, mediated the axon growth, also can rebuild the blood vessels, promote local the formation of new blood vessels, making neurotrophic improve, improve the survival of the nerve cells rate, is advantageous to prevent the secondary damage of patients with spinal cord tissue, improve the neuron axon regeneration function, reconstruction of nerve conduction function and alternative for myelination: medicine is generally believed that BMSC to the nerve cell phenotypic differentiation, table of neuronal specific marker protein, to replace damaged or death of nerve cells in the spinal cord; But studies have shown that if the number of differentiated cell too little, spinal nerve functional restoration of the phenomenon of solution of interpretation is difficult to use alternative cell differentiation, (3) phagocytosis: BMSC transplantation to the patients of spinal cord is damaged, can devour necrotic tissue, (4) inhibition against nerve regeneration of scarring bridge: BMSC can bridge in patients with SCI end, can make the work synapse formation; A BMSC transplanted to the site of SCI can provide a scaffold to promote axon regeneration and guide chemotactic axon forward growth. Studies have confirmed that the co-culture of BMSC and NSCs can induce the differentiation of stem cells into neurons for self-repair and promote the recovery of spinal nerve function in patients.

Injectable polymer nanoparticle (PNP) hydrogel platform, utilizing the polyvalent non-covalent interaction between modified biopolymers and biodegradable nanoparticles, has shown promise for the inclusion and delivery of human mesenchymal stem cells (hMSCs) based therapy due to its extensive differentiation ability and the production of therapeutic paracrine signaling molecules, *In vivo* studies of immune-capable mice showed that PNP hydrogel enhanced the retention of hMSC at the injection site and retained hMSCs locally for more than 2 weeks (Grosskopf et al., 2020). Of this study is based on HA and adhesion peptide PPFLMLLKGSTR, developed a kind of peptide modified stents synchrotron radiation micro computed tomography (CT) scanning measurement for the porous structure of 3D terrain features and internal perspective provides the train of thought in the process of *in vitro* three-dimensional culture, between the stent significantly improves the cell survival rate and adhesion of MSCs to grow, It was found to have a synergistic effect, and the composite implant had the most significant impact on the motor function score of the hind limb 2 weeks after the recovery of the injured spinal tissue with respective strength, namely the time when the secondary injury factors began to form (Li et al., 2017).

Induced pluripotent stem cells are derived from differentiated somatic cells after a certain genetic modification. They have many advantages of stem cells. They have the self-renewal capacity and differentiation potential like ESCs, avoiding immune rejection and ethical issues. IPSCs serve as a source cell that can be directly obtained from tissues and used in autologous transplantation, have become a hot spot in the field of stem cell research at home and abroad. Neurospheres derived from human iPSCs implanted in the damaged spinal cord can survive and migrate there and transform into neuronal astrocytes or oligodendrocytes. Neurons differentiated by hiPSC-NSs can establish functional synaptic connections with host neurons, increase the expression of NGF, promote the regeneration of blood vessels in the injured area and the formation of myelin sheaths and neural precursor cells. After implantation of iPSCs, the injured spinal cord microenvironment can be improved by promoting the expression of VEGF, thus indirectly increasing the number of its own neurons and promoting the regeneration of distal spinal axon.

Embryonic stem cells are highly undifferentiated cells with totipotency and the ability to differentiate into adult animal cells due to their high degree of undifferentiation. Since ESC is a totipotent cell that can differentiate into all tissue cells, including neurons, it is also currently considered as a seed cell with great potential for neurodegenerative diseases. ESCs from blastocysts, and has a developmental totipotency and can develop into three layer derived the ability of cervical SCI model, from the brain and spinal cord tissue of the embryonic spinal cord or spinal cord neural progenitor cells as a graft, transplanted cells can survive and differentiate into neurons cells, make the movement function of SCI model obviously improve. it is worth noting that the chest and amyotrophic lateral sclerosis, SCI model in the transplantation methods effect is good; In clinical practice, NSCs from embryonic tissue were transplanted to the site of cervical and thoracic SCI.

### Polymer Scaffolds Growth Factor Composite Platform

Growth factors play an important regulatory role in the development and damage repair of the central nervous system. After SCI, the deficiency of endogenous GFs secretion and generation is another important factor inducing difficulty in nerve regeneration. Therefore, supplementing exogenous GFs will greatly promote SCI repair. However, due to its short half-life in the organism, it needs to be administered for many times, and the traditional intramuscular injection is difficult to pass the blood-spinal cord barrier, nor can it improve the local drug concentration in the damaged area, so its application in the field of SCI repair is severely limited. In this context, the emergence of engineering three-dimensional stents provides good prospects. In addition to combining 3D scaffolds with transfected stem cells as described above, directly combining GFs to form functional engineering 3D scaffolds also shows good therapeutic effects.

**FIGURE 4:**
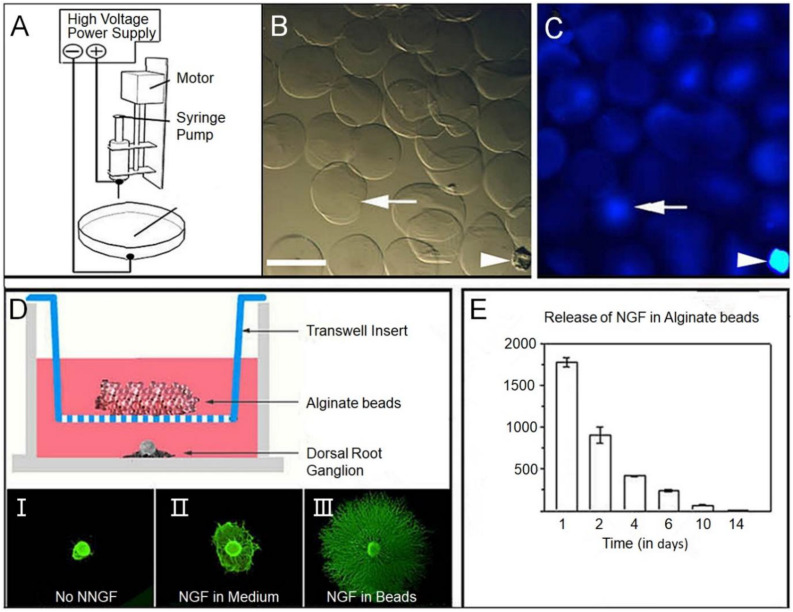
**(A)** Schematic diagram of the alginate electrospray apparatus. **(B)** Micrograph of alginate beads showing uniform diameter. **(C)** Fluorescence photography, beads were showing fused luciferin conjugated secondary antibodies (blue). The white arrow points to the same bead, and the arrow points to the fragment for spatial reference. **(D)** Bioactivity of nerve growth factor (NGF) binding alginate microspheres on dorsal root ganglion (DRG) growth was studied by the Transwell culture test. In this experiment, beads were placed into Transwell inserts in Wells covered with E15 rat DRG. **(E)** The excretion time of alginate microspheres was prolonged by ELISA; SEM mean values of repeated culture were given. Reproduced with permission from Colello et al. (2016).

Various types including NGF, neurotrophic protein 3 (NT-3), BDNF, fibroblast growth factor (FGF), Glial cell derived neurotrophic factor (GDNF) and insulin-like GF have been used in combination with tissue engineering scaffolds for repair of SCI. NGF is one of the most important bioactive molecules in the neurotrophic factor family, NGF is not only abundant in the nervous system, but also widely distributed in the non-nervous system. NGF plays an important role in promoting the regeneration and neuroplasticity after the growth and demyelination of axons in the survival differentiation of neurons. In this study, a PLGA porous scaffold was prepared by phase conversion method, and the scaffold surface was modified with polydopamine (PDA) as substrate, and then the NGF was adsorbed to obtain the PDA-PLGA/NGF scaffold, PDA modification can significantly improve NGF adsorption capacity and provide continuous release of NGF PDA-PLGA/NGF scaffold can not only enhance NSC proliferation and neuronal differentiation *in vitro*, but also promote the recovery of SCI *in vivo* (Pan et al., 2018). Electrospinning can be loaded with drugs for nutritional support and inflammation suppression, providing biochemical clue support for spinal cord repair (Figure 4; Colello et al., 2016).

Glial cell derived neurotrophic factor was isolated from the rat glioma cell line containing a 134 amino acid residues with dimers proteins, the molecular weight is 33∼35 kD, it is widely expressed in the central nervous system, and the expression of after puberty and damage increased GDNF in the neurons to protect glial scar reconstruction axon regeneration and sprout and then play an important role in myelination. GDNF can save the apoptosis of neurons after SCI. GDNF gene therapy with adenovirus vector can maintain the regeneration of neuron fibers and promote the recovery of motor function of the hind limb in spinal cord contusion. GDNF is a kind of neurotrophic factor, which plays an important role in neurotrophic aspect. GDNF can protect neurons from dying and necrosis, and it has a bidirectional regulation mechanism.

Brain-derived neurotrophic factor is a small molecule dimer with alkaline secretion that has been isolated and purified from the pig brain. BDNF plays an important role in promoting neuronal survival and proliferation against neuronal apoptosis. It also stimulates neurite growth and protects axonal excised neurons.

Basic fibroblast growth factor (bFGF) is a kind of neural protection because of the present study more, it is mainly distributed in the pituitary adrenal retina nerve tissue and placental tissue, such as the highest content of pituitary the main biological effects including bFGF promote tissue regeneration of the wound healing and tissue repair, and can participate in nerve regeneration, repair hair and in the central nervous system damage in has the vital significance. In addition, bFGF also plays a protective role in protecting the microenvironment from SCI by inhibiting nitric oxide toxicity and reducing free basal formation of stable cell calcium and magnesium ionized water.

Will be equipped with NT-3 of the chitosan carrier inserted in the ridge pulp crosscutting clearance, the results show that with the Nestin, Tuj1 and NeuN positive cells in nerve tissue bridging the transection clearance, but also to sports and somatosensory evoked potential and hind leg movement significantly improve further confirmed that the chitosan carrier implants by causing endogenous neurogenesis to promote the formation of nerve tissue, improve the recovery of function.

### Polymer Scaffolds Small Molecule Drugs Composite Platform

Small molecule drugs play a vital role in the treatment of various diseases such as anti-tumor, nerve damage, tissue repair, are a popular choice for clinicians and patients. Many small molecule drugs have been developed to promote the repair of the spinal cord. Such as cortisol hormones, immunomodulators, and traditional Chinese medicine extracts such as curcumin and ginsenosides. However, recent literature has suggested that the simple application of MP in the treatment of SCI cannot achieve satisfactory results, and the serious complications brought by the systemic high-dose application of MP cannot be ignored. 3D engineering scaffolds have unparalleled advantages as drug carriers and are an effective alternative.

Methylprednisolone is the only drug approved by the FDA to treat SCI. MP is one of the types of corticosteroid hormone, can inhibit lipid peroxidation and edema after injury and so on support of the inflammatory response in the SCI repair but lately have been put forward, the application of pure MP for the treatment of SCI is not satisfactory, and systemic high-dose serious complications brought by the application of MP also not allow to ignore. Therefore, the use of stent-loaded MP for local release for the treatment of SCI is widely studied. MP was encapsulated in degradable PLGA NPs and embedded into agarose hydrogel to locate contusions, When through the hydrogel nanoparticles system conveying, MP into the SCI, and spread in the injury of spinal cord within 2 days about 3 mm to 1.5 mm deep and in addition, the local MP significantly reduced inflammation early delivery in the spinal cord contusion injury (Chvatal et al., 2008).

Minocycline is a second-generation semi-synthetic tetracycline antibiotic, which plays an anti-inflammatory role mainly by binding to the ribosomal 30S subunit to prevent the extension of peptide chain and inhibit bacterial protein synthesis. In addition to its antibacterial effects, minocycline has been used in experimental models of a variety of neurological diseases, including stroke, traumatic brain injury, neuropathic pain, Parkinson’s disease, Huntington’s disease, amyotrophic lateral sclerosis, Alzheimer’s disease, multiple sclerosis, and SCI. Minocycline can show various effects such as anti-inflammatory, anti-oxidation, and anti-apoptosis in the treatment of SCI to inhibit secondary injury. We developed a PSA based nano drug delivery system of minocycline (PSM), preparation of PSM for collaborative treatment in SCI in both *in vivo* and *in vitro* has obvious anti-inflammatory and neuroprotective activity PSM management can significantly prevent neurons and myelin damage, reduce the formation of glial scar, the recruitment of the lesion site endogenous NSCs, promote the regeneration of the neurons and the extension of the glial scar long axons, which largely improve movement function of SCI in rats and imposed a superior therapeutic effect (Wang X. et al., 2019).

As an important anti-tumor drug, paclitaxel can affect the dynamic balance between a and B tubulin dimers and tubules, promote the assembly of tubules into microtubules, stabilize the formed microtubules, and block cells during mitosis. Paclitaxel can reduce injury after SCI in rats inflammatory reaction, reduce tissue damage and less reactive glial, narrow glial scar, improve the function of rat hind leg movement, prompt application of paclitaxel therapy may be helpful in rats after SCI effective pathways to rebuild low doses of the drug did not affect after SCI induced cell death as a result, paclitaxel in low doses, by standing alone outside of the cell proliferation or apoptosis mechanism to reduce fibrous scar formation of taxol can inhibit astrocyte proliferation and glial scar formation, have a protection effect on chronic SCI. We designed a dual administration system consisting of minocycline (MH), a neuroprotective drug, and paclitaxel (PTX), a neuroregenerative drug, to enhance tissue regeneration in rat SCI halfcut models, to this end, PTX-encapsulated PLGA microspheres were combined with MH into alginate brine gel. The continuous release time of MH and PTX in alginate hydrogel was over 8 weeks, Histological evaluation showed a reduction in inflammation after 7 days of dual-drug therapy. In addition, rats on the dual-drug regimen had reduced scar tissue and increased neuronal regeneration after 28 days. Over time, the animals receiving the dual–drug regimen showed rapid and sustained improvement in function compared with other groups (Nazemi et al., 2020). Taxol was added to electrospinning poly(*L*-lactic acid) (PLLA) microfibers, and it was confirmed that taxol released from well-arranged electrospinning microfibers promoted neurite extension in growth-friendly and growth-inhibiting environments (Roman et al., 2016).

Curcumin is a natural polyphenolic compound extracted from the Traditional Chinese medicine curcumin, which has shown good efficacy in the repair of SCI. Curcumin can inhibit the neuritis reaction, reduce the formation of glial scar, reduce free radical release and lipid peroxidation in local nerve tissues and it can also reduce neuronal apoptosis and improve SCI microenvironment.

### Conclusions and Prospects

Spinal cord injury is one of the most damaging human pathologies, severely impacting the quality of life and the overall society. Since the pathology of SCI is robust and evolving in nature with the progressive interplay between different molecular and biochemical cascades, therapies designed to control only one aspect of these cascades cannot modulate and control the parallel axes that indirectly or directly influence the selected axis. The first challenge is to prevent the progression of cascades of subsidiary injury. The second challenge is to regenerate the injured spinal cord and to restore neuronal connectivity. Therefore, we recommend tailoring the incorporation of biological and engineering approaches derived from the identified interplay between their respective mechanisms throughout recovery from SCI. In this regard, polymer-based therapy can play an essential role as it can hence neuroprotection and promote axon regeneration. Especially, polymer-based therapies can play a dual role in neuroprotective, as well as neurogenerative therapeutics and acting as a scaffold for tissue engineering and cell-based treatments to enhance rejuvenation. Additionally, the polymer, in combination with proliferation factors and the neuroprotective agent, could prospectively promote rejuvenation and functional recovery after SCI.

Current polymer-based therapies have shown therapeutic prospectives in animal models, but their potential to mediate clinical improvements after SCI remains elusive. From our perspective, we recommend that combinatorial therapy paradigms primarily targeted to the chronic stage are further pursued in the field since there are millions of people globally suffering from SCI that are well past their moment of injury.

## Author Contributions

MW, YW, and WQ conceived the article. WQ wrote the article with the support from BC. WS, HT, XO, and XZ revised the article and approved the final version. All authors contributed to the article and approved the submitted version.

## Conflict of Interest

The authors declare that the research was conducted in the absence of any commercial or financial relationships that could be construed as a potential conflict of interest.
